# Detection of West Nile Virus in Oral and Cloacal Swabs Collected from Bird Carcasses

**DOI:** 10.3201/eid0807.020157

**Published:** 2002-07

**Authors:** Nicholas Komar, Robert Lanciotti, Richard Bowen, Stanley Langevin, Michel Bunning

**Affiliations:** * Centers for Disease Control and Prevention, Fort Collins, Colorado, USA; † Colorado State University, Fort Collins, Colorado, USA; ‡ United States Air Force, Bowling Air Force Base, Washington, DC, USA

**Keywords:** West Nile virus, *Flaviviridae*, flavivirus, fecal shedding, virus isolation, viral RNA detection, bird mortality

## Abstract

We evaluated if postmortem cloacal and oral swabs could replace brain tissue as a specimen for West Nile virus (WNV) detection. WNV was detected in all three specimen types from 20 dead crows and jays with an average of >10^5^ WNV PFU in each. These findings suggest that testing cloacal or oral swabs might be a low-resource approach to detect WNV in dead birds.

Since 1999, surveillance of bird deaths has become a standard epidemiologic method for detecting the spread and continued presence of West Nile virus (formal name: *West Nile virus* [WNV]) transmission throughout the eastern United States (1). In 2000 alone, approximately 13,000 bird carcasses were tested for WNV (2). Substantial resources are required to accomplish the tasks associated with this novel type of arbovirus surveillance: transport of the avian carcasses to a laboratory (often distinct from the microbiology laboratory where diagnostic testing will be performed), organ removal, tissue maceration and clarification, and testing of tissue homogenates. We considered ways to simplify these tasks.

Given that birds with acute WNV infection frequently shed the virus in cloacal or oral cavities (3–6) and that we have detected very high WNV titers (e.g. 10^6^ PFU) on cloacal and oral (nasopharyngeal) swabs of corvid^1^ and other passerine birds with experimentally induced, acute WNV infections (N. Komar, unpub. data), we hypothesized that cloacal swabs or oral swabs from carcasses could replace brain samples, the preferred tissues to test for WNV infection in corvid carcasses (7).

## The Study

We collected postmortem specimens from 20 corvids, including 12 American Crows (*Corvus brachyrhynchos*), 4 Fish Crows (*Corvus ossifragus*), and 4 Blue Jays (*Cyanocitta cristata*), that had died (or in one case had been euthanized after being moribund for 24 hours) after experimental infection with the New York 1999 strain of WNV. (The modes of infection^1^, sampling protocol, and resulting pathogenesis will be described separately.) Brain and other organs were harvested, and postmortem cloacal and oral swabs were collected (using standard cotton- or Dacron-tipped applicators) in 0.5-mL physiological buffer containing antibiotics, within 24 hours of death. All specimens were frozen at -70°C until assayed for virus content by Vero plaque assay and for WNV-specific RNA by TaqMan reverse transcriptase–polymerase chain reaction (RT-PCR), as previously described (8).

 We detected WNV RNA in all postmortem brain tissue samples as well as cloacal and oral swabs. Infectious WNV particles were detected in all but one specimen, a cloacal swab taken from a Fish Crow. Viral titrations and quantitative TaqMan RT-PCR indicated that the concentrations of WNV averaged >10^5^ in all three specimen types (Table).

**Table:** Mean logarithmic titers of West Nile virus (WNV) infectious particles, determined by Vero plaque assay and TaqMan reverse transcriptase–polymerase chain reaction^a^

	Specimen type (Mean Vero log PFU [range]/Mean TaqMan log PFU equivalents [range])		
Species	Brain	Oral swab	Cloacal swab
American Crow	8.2 [5.9–8.8]/7.1 [5.3–7.7]	7.3 [4.1–7.7]/6.6 [4.6–7.1]	6.4 [3.8–7.4]/6.9 [6.1–7.3]
Fish Crow	6.6 [4.1–6.9]/5.8 [4.8–6.2]	7.0 [1.4–7.6]/6.1 [3.2–6.7]	6.8 [<0.4–7.4]/6.0 [2.3–6.6]
Blue Jay	8.0 [7.3–8.2]/6.3^b^ [6.2–6.3]	7.1^b^ [5.3–7.4]/5.7^b^ [4.4–6.0]	5.8 [3.0–6.3]/6.7^b^ [5.6–7.0]

^a^In postmortem samples of brain tissue (1 cm^3^), and oral and cloacal swabs for 12 American Crows, 4 Fish Crows, and 4 Blue Jays experimentally infected with the New York 1999 strain of WNV. ^b^This value determined from only two birds.

## Conclusions

Avian mortality surveillance for WNV targets fresh carcasses (generally dead <24 h), especially corvids, for detection of infectious virus particles or RNA in brain or other viscera. We have shown that postmortem oral and cloacal swabs, in addition to brain, are effective samples to collect for WNV detection in experimentally infected corvids. A potential implication of these findings, pending field trials using corvids and other species routinely collected as part of avian mortality surveillance, is that WNV may be detected by simply collecting swabs from carcasses and forwarding the swabs (frozen) to a virology laboratory for testing. Eliminating multiple steps currently necessary for WNV testing of bird carcasses may conserve valuable public health resources and reduce the risk of exposure for laboratory personnel.

## Footnotes

1 Pertaining to the family Corvidae, including crows, jays and magpies.

## References

[1] Eidson M, Komar N, Sorhage F, Nelson R, Talbot T, Mostashari F, Crow deaths as a sentinel surveillance system for West Nile virus in the northeastern United States, 1999. Emerg Infect Dis. 2001;7:615–20. 11585521 10.3201/eid0704.010402 PMC2631775

[2] Marfin AA, Petersen LR, Eidson M, Miller J, Hadler J, Farello C, Widespread West Nile virus activity, eastern United States, 2000. Emerg Infect Dis. 2001;7:730–5. 11585539 10.3201/eid0704.010423 PMC2631748

[3] Senne DA, Pedersen JC, Hutto DL, Taylor WD, Schmitt BJ, Panigrahy B. Pathogenicity of West Nile virus in chickens. Avian Dis. 2000;44:642–9. 10.2307/1593105 11007013

[4] Swayne DE, Beck JR, Zaki S. Pathogenicity of West Nile virus for turkeys. Avian Dis. 2000;44:932–7. 10.2307/1593067 11195649

[5] Langevin SA, Bunning M, Davis B, Komar N. Experimental infection of chickens as candidate sentinels for West Nile virus. Emerg Infect Dis. 2001;7:726–9. 11585538 10.3201/eid0704.010422 PMC2631771

[6] Swayne DE, Beck JR, Smith CS, Shieh WJ, Zaki SR. Fatal encephalitis and myocarditis in young domestic geese (*Anser anser domesticus*) caused by West Nile virus. Emerg Infect Dis. 2001;7:751–3. 11585545 10.3201/eid0704.010429 PMC2631765

[7] Panella NA, Kerst AJ, Lanciotti RS, Bryant P, Wolf B, Komar N. Comparative West Nile virus detection in organs of naturally infected American Crows (*Corvus brachyrhynchos*). Emerg Infect Dis. 2001;7:754–5. 11592255 10.3201/eid0704.010430 PMC2631762

[8] Lanciotti RS, Kerst AJ, Nasci RS, Godsey MS, Mitchell CJ, Savage HM, Rapid detection of West Nile virus from human clinical specimens, field-collected mosquitoes, and avian samples by a TaqMan reverse transcriptase-PCR assay. J Clin Microbiol. 2000;38:4066–71. 11060069 10.1128/jcm.38.11.4066-4071.2000 PMC87542

